# Innovative MRI Techniques in Neuroimaging Approaches for Cerebrovascular Diseases and Vascular Cognitive Impairment

**DOI:** 10.3390/ijms20112656

**Published:** 2019-05-30

**Authors:** Lorenzo Carnevale, Giuseppe Lembo

**Affiliations:** 1 IRCCS Neuromed, Department of AngioCardioNeurology and Translational Medicine; 86077 Pozzilli, Italy; lorenzo.carnevale@neuromed.it; 2 Department of Molecular Medicine; University of Rome “Sapienza”, 00185 Rome, Italy

**Keywords:** cerebrovascular diseases, dementia, vascular cognitive impairment, brain magnetic resonance imaging

## Abstract

Cognitive impairment and dementia are recognized as major threats to public health. Many studies have shown the important role played by challenges to the cerebral vasculature and the neurovascular unit. To investigate the structural and functional characteristics of the brain, MRI has proven an invaluable tool for visualizing the internal organs of patients and analyzing the parameters related to neuronal activation and blood flow in vivo. Different strategies of imaging can be combined to obtain various parameters: (i) measures of cortical and subcortical structures (cortical thickness, subcortical structures volume); (ii) evaluation of microstructural characteristics of the white matter (fractional anisotropy, mean diffusivity); (iii) neuronal activation and synchronicity to identify functional networks across different regions (functional connectivity between specific regions, graph measures of specific nodes); and (iv) structure of the cerebral vasculature and its efficacy in irrorating the brain (main vessel diameter, cerebral perfusion). The high amount of data obtainable from multi-modal sources calls for methods of advanced analysis, like machine-learning algorithms that allow the discrimination of the most informative features, to comprehensively characterize the cerebrovascular network into specific and sensitive biomarkers. By using the same techniques of human imaging in pre-clinical research, we can also investigate the mechanisms underlying the pathophysiological alterations identified in patients by imaging, with the chance of looking for molecular mechanisms to recover the pathology or hamper its progression.

## 1. Background

As life expectancy has increased, a progressive burden on the healthcare system is imposed by the treatment and caregiving of cognitively impaired and demented patients. To face this emergency, the World Health Organization (WHO) has declared all non-communicable diseases a threat to the world population [1]. The estimates state that 35.6 million people are currently affected by dementia and a 3-fold increase will be registered by 2050 [1]. Recent studies have demonstrated that while neurodegenerative processes underlie many cases of dementia like Alzheimer’s disease, in a large number of cases, concomitant vascular pathology has been identified [2,3]. To support the pivotal role of the vasculature in cognitive impairment, many studies have investigated the pathophysiology of the “neurovascular unit”. This definition encloses all of the functional cell–cell interactions necessary to develop and maintain the vasculature in the brain and ensure the energy supply to neurons. A malfunction of this domain can originate from vascular pathologies, but inevitably reflects on neural functioning. While the most accepted vascular cognitive impairment (VCI) definition states that VCI is “a syndrome with evidence of clinical stroke or subclinical vascular brain injury and cognitive impairment affecting at least one cognitive domain” [4], damage or loss of adequate functioning of the neurovascular unit can nonetheless affect cognition. Hence, the need for a new way to explore the brain and its vasculature to identify characteristic patterns of alterations that can predict the onset of cognitive impairment and cast light on its pathophysiological origin.

To date, the best available tool to explore the human brain is magnetic resonance imaging (MRI). Originating around fifty years ago, this technology leverages the property of hydrogen nuclei, which when stimulated by radiofrequency pulses, resonate with the magnetic field where they are immersed, allowing the analysis of different tissues. By reading the signals emitted during the resonance, we can obtain the time needed for a particular tissue to return to a steady state on the longitudinal component or on the transverse one, which is the T1 relaxation time and T2 relaxation time, respectively. The images are obtained by weighting one of the two components, emphasizing the contrasts between tissues with different T1 or T2 relaxation times. During the past decades, MRI techniques have evolved from methods that allow images of internal tissues of the patients to be obtained to techniques capable of providing functional insights of the biological systems under examination. 

The support of various MRI techniques can be a fundamental addition to clinical practice to specifically characterize and diagnose different forms of cognitive impairment originating from vascular pathologies. On this note, a modern and quantitative approach can be instrumental to extrapolate effective biomarkers for clinical and modern computer-driven analyses.

## 2. Structural MRI to Quantify Morphological Alterations

The first applications of brain MRI were developed to understand and analyze the morphological alterations induced by various pathologies impacting on white and grey matter [5,6]. The first approach aimed to obtain a segmentation of the brain and a parcellation of the cortical areas by hand or by using specialized software [7,8]. The data obtained from these elaborations were used to characterize the neurodegenerative processes and related to the affected physical areas with the associated cognitive functions [9]. 

Another strategy that allows for the investigation of differences in gray matter distribution and density is voxel-based morphometry (VBM) [10]. This technique eliminates the intrinsic variability of brain dimensions in morphological analysis by co-registering the images in a common space for all of the subjects in a study, and then, after mathematical operations such as smoothing and image normalization, a voxel-wise statistical comparison of the gray matter is performed to highlight the differences between different groups in the study [11].

While atrophy or deformation of gray matter integrity are visualizable and measurable through macrostructural evaluations, white matter lesions are often injuries at the microstructural level, thus not measurable in macroscopic morphology. The development of new techniques specific for white matter injury evaluation has paved the way for the analysis and identification of one of the most important markers of cerebrovascular damage in the brain: the white matter hyperintensities (WMH). The T2-FLAIR sequence (T2-Fluid Attenuated Inversion Recovery) [12] is useful to highlight regions of T2 prolongation in the white matter, corresponding to regions of increased water content with respect to normal white matter. In this kind of sequence, areas of hyperintensity represent a region where the white matter is undergoing a process of demyelination or axonal loss. In general, these alterations are the main evidence of small vessel disease (SVD) progression, even though they can correspond to different pathological states [13].

Characterization of WMH was first qualitative, with a grading system based on the appearance and position of the identifiable lesions [14], then, with the progress of computer-aided diagnosis (CADx) systems and improvements in the computer vision field, we can now absolutely quantify the volume of white matter lesions [15,16]. This improvement is fundamental to defining the absolute and quantitative biomarkers that could help in better evaluating and predicting the onset of VCI in the population at risk. 

Another structural hallmark of SVD is the alteration of the Virchow–Robin or perivascular spaces (PVS) [17]. The multifaceted role of the PVS in maintaining the Blood Brain Barrier (BBB) equilibrium and brain homeostasis makes the study of its alterations of utmost importance to identify markers of damage associated with cerebrovascular pathologies [18]. Similarly to WMH, it is possible to evaluate the PVS alterations both with a semi-quantitative grading system [19] or with quantitative automatic methods [20,21]. The latter category produces quantitative measures, which can contribute to an estimate of the global cerebrovascular risk, together with other brain measurements.

## 3. Diffusion Imaging to Evaluate White Matter Integrity

Diffusion weighted imaging (DWI) is a MRI technique that locally modulates the static magnetic field. This allows us to evaluate the intensity of the spontaneous diffusion of water along that direction. To obtain a comprehensive model of the Brownian motion of the water in the different tissues of the brain, we can combine multiple independent directions of the magnetic field in subsequent scans [22]. By concatenating the resulting various images, we can obtain a four-dimensional image, where three dimensions represent the spatial information and the fourth dimension represents the different orientation of the magnetic field in each scan. The use of more than six directions of magnetic field lets us apply a tensorial model to each voxel of the image to characterize the preferential direction in which the water diffuses [23]. This imaging technique, diffusion tensor imaging (DTI), is a powerful technique that can be used to obtain a parametric representation of the microstructural organization of structures like the myelinated axonal fibers composing the white matter. The main parameters obtained from DTI are fractional anisotropy (FA) and mean diffusivity (MD), axial diffusivity (AxD), and radial diffusivity (RD). The first represents the rate of directionality of water diffusion in a single voxel along the favored direction (i.e., along the axon direction in a voxel of white matter), and the second represents the mean intensity of diffusion in a voxel, often correlated with the water content of that voxel [24]. The last pair of parameters express the intensity of diffusion along the favored direction of diffusion (AxD) and along the orthogonal plane to it (RD), respectively.

Once a complete modeling of water diffusion in the brain has been obtained, it is possible to segment regions where the diffusion direction is coherent, and then reconstruct the connections between different brain areas in this way [25]. This technique, referred to as tractography, allows microstructural information of different brain areas to be obtained, selecting them only on the basis of spatial location, but also in relation to the anatomical connections [26].

With the evolution of MRI scanners, DTI has been perfected to have increased spatial and angular resolution, allowing better discrimination of fibers in smaller regions and reconstructing them with higher precision by fitting the model to scans obtained by modulating the field at different intensities (Multi-Shell DTI) [27]. The evolution of imaging sequences has been followed by progression in the analytical models applied to the resulting data to better characterize the connections, which takes into account the limits of the imaging modality such as the impossibility to visualize two different directions of diffusion guided by crossing white matter fibers in a single voxel. Modern computational probabilistic diffusion models and probabilistic fiber tracking let us infer these connections from the data and provide us with a comprehensive representation of the different physical connections in the brain [28,29].

The investigative power provided by the use of DTI in assessing the status of white matter microstructure is a powerful tool in the hand of clinicians to understand the impact of vascular challenges on the brain characterizing the pathophysiological process underlying the injury. Briefly, a decrease in FA can usually be considered an index of white matter fascicle disorganization due to demyelination or axonal degradation. On the other hand, MD is a less specific indicator that is often associated with the damage of neuronal membranes [24]. One of the most common approaches used to analyze DTI is the use of tract-based spatial statistics (TBSS) [30], which acquires a group of images to a common space, projects the mean FA or MD values to the skeletonized profile of the white matter, and then computes cross-subject voxelwise statistics to assess the differences in some regions between various conditions. By using this method, it became possible to assess differences in the spatial location of signs of damage that discriminate between vascular dementia (VaD) and pure AD [31]. 

Implementing fiber-tracking approaches allows a per-patient segmentation and extraction of the values of diffusivity [32,33,34]. As an example, this approach makes it possible to link the increased risk in all-cause mortality and stroke with alterations of FA and MD, independently from other white matter damage such as WMH volume [33,35]. In a similar way, it is possible to relate the damage derived from specific clinical conditions like hypertension with FA alterations in specific tracts associated with the decline of performance in cognitive domains driven by those tracts [34]. Other approaches have tried to further increase the sensitivity of DTI to tackle rare diseases such as cerebral autosomal dominant arteriopathy with subcortical infarcts and leukoencephalopathy (CADASIL), a genetic condition causing a spontaneous form of SVD evidenced by leukoaraiosis. After the identification of the tracts, their skeletonization has been useful to avoid signal interferences from cerebrospinal fluid and increase the sensitivity of the newly obtained DTI parameters (PSMD, peak width of skeletonized mean diffusivity). PSMD alterations have been associated with CADASIL or sporadic SVD, but not to AD [36].

DTI processing lacks a well-established gold-standard that can be adopted in the process of a clinical trial, thus confining this technique to independent research projects or methodological research. This limitation comes mainly from the availability of dozens of diffusion models to reconstruct the data, algorithms to track the fibers, and software to carry out the computation. One of the aims of the radiological and computational neuroimaging community should be to compare different combinations of operations to create a set of tools to be proposed as a gold-standard to extract diffusion parameters and fiber bundles reconstructions, from the perspective of being usable as biomarkers for clinical practice. 

## 4. Functional MRI to Highlight Networks of Neurons

Functional MRI is an imaging technique that exploits the neurovascular mechanism called “functional hyperemia” whereby ongoing brain regional activation recruits increased blood oxygenation in that specific region [37]. The most common implementation of fMRI is a T2*-weighted sequence of images with a weighting approximatively equal to the tissues T2* relaxation time and with a scan time lower than 5 s to correctly sample the hemodynamic response function (HRF).

Thus, the analysis of blood oxygen level-dependent (BOLD) signals allows for the indirect measurement of regions of brain activation. The first applications of this technique were implemented to actively map brain areas activated in response to various stimuli to associate regions of neural activity to specific functions such as motor control or visual stimuli processing [38,39]. An evolution of this analytic paradigm has been obtained with the breakthrough discovery of regions with synchronized activity, independent from the stimulus provided to the patient under examination, i.e., in a resting state condition (rs-fMRI) [40,41]. By exploiting this strategy, a consistent pattern of synchronized activation in healthy subjects has been shown. These patterns, called functional networks, have been associated with the cerebral function driven by those synchronized regions. A pivotal study by Yeo et al. [42] analyzed the resting state fMRI sequences of 1000 healthy adults to obtain a parcellation of the gray matter. By clustering the voxels obtained according to the similarity of their BOLD signal over time, i.e., their functional connectivity, they represented either seven (coarse resolution) or 17 (fine resolution) functional networks.

Rs-fMRI image datasets are usually made of a high number of volumes acquired in succession, show low contrast between different brain tissues, and generally do not provide much information to the visual inspection. Many different approaches have been tested to analyze this kind of data, resulting in dozens of algorithms, parameters that can be extracted, and different approaches to obtain results from the sequences. This approach has generated a situation similar to DTI analysis, where no clear golden standard exists. Similarly, the analytical principles are driven by the solutions implemented in the major neuroimaging software suites, which have been adopted by international consortiums [43].

The first approaches tried to leverage the a priori knowledge of radiologists and relied on the identification of regions of interest (ROIs), which are the seed for searching areas of analogous activity. This technique, referred to as seed-based functional connectivity analysis, was used in the very first paper that identified and formalized the concept of a functional network [40]. With the evolution of mathematical models available to tackle high dimensional problems such as rs-fMRI and the improvement of computational capabilities, there has been a paradigm shift from imposing initial conditions (i.e., seed ROIs) to the data to extracting patterns by leveraging techniques that infer spatial and temporal organization of the brain activity directly from the raw timeseries. The main example of this approach is the independent component analysis, a blind signal separation that assumes that the signal acquired is the result of various spatio-temporal processes statistically independent between each other [44]. By extracting all of the different independent signals, we can reconstruct the various timecourses of specific brain regions, grouping them into maps representative of their spatial distribution. Another approach to the analysis of rs-fMRI datasets originated from Graph Theory, a mathematical set of tools that represent a system as a combination of nodes and edges. The nodes denote the data from our system, while the edges resemble the interactions established between two connected nodes. In the rs-fMRI, we can represent the different regions of the cortex as the nodes of our graph, and the similarity between their timecourses (the functional connectivity) as the edges between themselves. Once the network has been built, it is possible to extract different mathematical measurements such as the average path length of the graph to describe the connectivity inside the brain [45,46]. 

AD research was one of the first fields where the use of fMRI has brought major advancements to the understanding of the pathophysiological processes underlying the ensuing cognitive decline. A consistent body of work demonstrated that AD alters the functional connectivity in the default mode network (DMN), a network that contributes to the default function of the brain and is deactivated during cognitively active tasks [47]. After these first investigations, with the increase of available data and improvements in data quality, many projects have also focused on different functional networks and different degrees of cognitive impairment as well as considering mild cognitive impairment (MCI) as an intermediate condition [48,49], thus showing a widespread alteration of functional connectivity in AD patients. Other reports in the literature have focused their interest on functional network properties by performing graph theoretical analysis, evidencing different graph connection properties in patients stratified by the presence of white matter lesions and VaD [50,51].

In a very similar way to DTI, one of the biggest shortcomings of rs-fMRI is the absence of a gold standard acquisition and analysis pipeline. Since it is a technique that measures neuronal activity by reading the effects of neurovascular coupling, it would be of fundamental importance to better explain the pathophysiological alterations underlying the cognitive decline induced by cerebrovascular diseases. The identification of a set of metrics that allows for the evaluation of alterations in the functional connectivity in an objective and standardized way, should be an absolute priority to define a functional biomarker which can be, at the same time, sensible and specific for a large set of pathologies grouped under the definition of VaD and VCI.

## 5. Magnetic Resonance Angiography and Arterial Spin Labeling for Imaging of the Cerebrovascular Tree, from Large Vessels to Microcirculation

The emerging role of neurovascular coupling in the regulation of cognitive function and brain homeostasis [52] has shown the importance of also taking into account the vascular contribution in pathologies classically attributed to neuronal dysfunction (i.e., AD). In VCI and dementia, it is even more valuable to assess the alterations of the cerebrovascular tree. MRI lets us characterize and evaluate the integrity of both large vessels and small vessels through different techniques.

Magnetic resonance angiography (MRA) is the technique of choice to study large vessels of the cerebrovascular tree [53]. The most diffused MRA sequence implemented to do this exploits the Time-of-Flight (TOF) effect, which is the variation of signal intensity in the presence of flow between two regions with different magnetizations [54,55]. The technique consists of saturating the magnetization of a region to nullify the signal of stationary tissues and highlight the signal originating by blood flowing from slices with no magnetic saturation. In this way, we can achieve a complete angiography of a desired region without injecting contrast agents into the patient, thus minimizing risks and undesirable side-effects.

The clinical use of MRA has been mainly introduced for the identification of aneurysms, nonetheless it can also be effective for the diagnosis and monitoring of pathologies such as Moyamoya disease, where a massive alteration of the cerebrovascular tree can be identified by a contrast-free angiography technique. While Moyamoya disease does not imply a canonical form of VCI, several studies have associated it with similar conditions of cognitive dysfunction [56,57,58], suggesting that large vessel diseases can also be a risk factor for cognitive decline and VaD.

If angiography can be a powerful tool to investigate the roots of the cerebrovascular tree, with this kind of technique, it is impossible to directly inspect the cerebral capillaries and understand their function in regulating cerebral hemodynamics. To this aim, specific sequences can evaluate the degree of tissue perfusion in a quantitative way [59]. Arterial spin labeling (ASL) and subsequent variants (pseudo-continuous, continuous, pulsed ASL) allow for this property to be evaluated: the principle is to obtain a steady state image that can be used as the control image, then one or more images after the magnetic tagging of the blood [60]. The blood circulating through tissues alters the T1 relaxation time and by applying specific mathematical models, it becomes possible to obtain an absolute quantification of perfusion. Like angiography, the main strength of ASL techniques is the absence of radioactive or kidney-toxic contrasts, making these approaches ideal candidates to obtain perfusion measurements in healthy populations or in studies that include one or more follow up analyses.

The principal use of the ASL perfusion technique is to image perfusion of brain tissue to (i) analyze ischemia or vascular malformations that alter the global perfusion, (ii) evaluate the hypervascularization of tumoral tissues in high risk populations, and (iii) evaluate the entity of traumatic brain injury to predict the prognosis of the patients. In addition, recent studies have used ASL to search for patterns of alterations in cerebral perfusion and their link with neurodegenerative diseases and dementia [61,62].

The combined analytical potential given by MRA and ASL to assess structure and function can be an invaluable support in the characterization and design of imaging biomarkers that quantitatively characterize the integrity and function of the cerebrovascular tree in a direct way.

## 6. New Analysis Techniques to Leverage Multimodal Big Data: Machine Learning Applied to Neuroimaging

The technological evolution in the field of computing has made it possible for the rise of a new branch of research that has narrowed the distance between computer science and biomedicine [63]. The first exchange between the fields started with the need for a system that could help radiologists in analyzing high volumes of data originating from the mass screenings of the population. An example of this issue is the diagnosis and risk assessment of breast cancer [64,65], or the identification of melanomas [66]. CADx systems were developed and implemented in clinical routine to lessen the workload for radiologists and dermatologists with a pre-evaluation of the data generated by the screenings and highlight region of interests to be analyzed by clinicians.

Machine learning (ML) is a branch of computer science that develops systems capable of learning from data to classify them into different classes. Classification can be implemented with two different approaches: supervised or unsupervised classification. The former leverages a set of input data described by various features and assigned to a set of classes, then trains an analytical model to correctly classify the highest number of input samples to the correct classes [67]. The latter does not have any information on the class assigned to the input data, and instead tries to develop an analytical model to extract patterns, which is useful to group similar data in different clusters [68].

The development of CADx systems and their introduction in clinical practice has been possible thanks to innovations in research. These systems take the patient data as input and apply a trained analytical model to give as output an area of interest for the radiologist or a risk assessment to better guide the clinician in the diagnostic process, depending on the specific problem. CADx, or in general ML algorithms, can implement different strategies of classification, each one better suited for specific classes of problems. As an example, the most common classifier algorithms are decision trees [69], support-vector machines (SVM) [70], random forests [71], and deep neural networks [72].

At the beginning of the new century, the technological push in data management systems, imaging platforms, and technology paved the way to the sprouting of collective efforts to gather data and elaborate them in a new perspective, not only from a clinician point of view, but in a way that could be tackled by automated systems to generate knowledge. Projects like the Alzheimer’s Disease Neuroimaging Initiative (ADNI) [73], Human Connectome Project (HCP) [74], and Human Brain Project (HBP) [75] have created databases of thousands of subjects available for the analysis that have been acquired with cutting edge technology and state-of-art protocols to ensure data quality and reproducibility. 

These neuroimaging initiatives are often paired with efforts to standardize the analysis pipeline and data extraction to achieve quantitative measurements of the brain that are suitable for advanced analysis with ML algorithms. One of the greatest breakthroughs originating from this approach has been the fine parcellation of the brain cortex, leveraging information of function, architecture, topography, and connectivity [43]. In the field of dementias, great efforts have been made toward the prediction and risk assessment of AD and transition from MCI to AD [76,77,78,79].

## 7. MRI in the Clinical Practice: toward Better Healthcare through Imaging Insights

The use of MRI in the context of vascular dementia and AD diagnosis has increased over the years with improvements in technical and image processing. Nonetheless, very few efforts have been directed toward a comprehensive characterization of the complex system of neurovascular functioning and cross talk between the cerebral vasculature and tissues. Even in AD, only greater efforts by consortiums like ADNI have produced multimodal data directed at achieving a global understanding of the pathology, thus directly impacting on patient management. The consortium produced has more than 1700 scientific publications, which have greatly advanced the understanding of the basis of AD and empowered clinicians with tools to aid them in the management and staging of high-risk patients [77,80,81] and with systems that can predict the transition from MCI to AD. Other initiatives applied to VCI have sprouted more recently, which have placed great effort into mapping the white matter lesions and the symptoms associated with their position and extent [82], with a particular attention on the data harmonization between different centers of the consortium and the unification of image processing protocols to grant reliable and repeatable results across different technical setups. 

## 8. Novel Imaging Strategies in Experimental Models: A Translational Approach from Bench to Bedside

The technological advances in electronics and miniaturization of the early nineties paved the way to affordable and industrialized MRI scanners for small animals. In preclinical MRI, the biggest challenge was the need for increased resolution to discriminate the smaller details of the rodent anatomy. To do this, it was necessary to increase the magnetic field and to push the gradient system and control the electronics at the limits of technological capabilities. Toward this aim, MRI scanners with a typical static magnetic field between 7 and 11.7 Tesla were developed, therefore combining high performance gradients with a smaller bore (between 16 and 40 cm), hence resulting in micrometric resolutions often with a high signal-to-noise ratio. It is worth noting that this increase in resolution and signal-to-noise ratio comes at a cost: the relaxation times of organic tissues in a 3 Tesla magnetic field are different from the relaxation times of the same tissues subjected to higher fields, like 7 Tesla [83]. This has forced researchers to set up a customized set of image contrasts to maximize the information that can be obtained from murine imaging, thus implementing all of the techniques discussed in the previous paragraphs.

The application of MRI to murine models of cognitive decline has proven to be a powerful tool to investigate the magnetic properties of various regions of the mouse brain in vivo, giving the chance to parallel the MRI findings to histological properties of analyzed regions. This kind of approach allows following anatomical variations such as the longitudinal analysis of brain atrophy as well as leveraging automated or semi-automated segmentation pipelines [84,85]. Despite the small dimensions and the substantial difference when compared to human white matter, DTI has also been used extensively to investigate the connections established between different areas of the brain. These studies have led to a complete tractographical mapping of the connections in the mouse brain [86] and to the assessment of detrimental effects on fiber structure and density in models of cognitive decline [87].

While the application of task-based fMRI is unfeasible in animal models, rs-fMRI has been implemented and used to investigate areas of synchronized activation in the anesthetized animal [88], providing insights complementary to the ones obtained by mapping physical connections and not achievable with ex vivo histological techniques. The implementation of this technique in mice is a technical challenge due to the complexity of the signals analyzed. In addition, different combinations of anesthesia and breathing conditions can also substantially affect the findings and need to be considered when discussing the obtained results (for a comprehensive review on mouse functional networks and animal handling, see [89,90]). As a tool for predicting pathological alterations, murine models of AD-like cognitive decline have been investigated with rs-fMRI, demonstrating the correlation between an increase of brain functional connectivity and the onset of tau pathology [91], suggesting that rs-fMRI can be an effective strategy to highlight the functional dysregulation correlated with cognitive decline [92].

In the study of murine models of cognitive decline, the evaluation of cerebral blood volume, cerebral blood flow, and perfusion has always been of great interest. While technologies like intracranial laser doppler for small animals have been available for decades, MRI has brought perfusion analysis to a completely new horizon. The imaging protocols let us investigate the integrity of the BBB, one of the main targets of molecular investigation aimed at clarifying the vascular contribution to cognitive decline, in the whole murine brain by adding fundamental spatial information to clearly identify the boundaries of altered microcirculation. Techniques that evaluate perfusion, cerebral blood volume, and BBB permeability [87,93] can be paired with genetic or experimental models of cognitive impairment and to mechanistic experiments to elucidate the pathophysiological processes involved in vascular and neurodegenerative diseases.

The use of MRI in preclinical research is an invaluable tool for adding both temporal or spatial dimension of analysis (i.e., longitudinal structural assessments or spatial blood flow measurements) to the functional study of the brain, giving insights about the topological organization of the functional networks. Moreover, a crucial aspect is the possibility of exploring, at a histological and mechanistic level, the damage or markers evidenced by MRI. In human studies, the only possibility is to perform autoptic studies or bioptic tissues (i.e., in tumor biopsies), thus limiting the possibility of understanding the mechanisms underlying the diagnostic image.

## 9. Conclusions and Perspective

The tight entwinement between neurons and the cerebrovascular tree suggests that cognitive impairment and neurological disorders can be often of mixed etiology. Thus, VCI and VaD need to be thoroughly characterized for their structural, microstructural, functional, and vascular pathological phenomenology. MRI can be a potent tool to investigate these characteristics together, with the aim of better discriminating the vascular component in cognitive impairments of mixed etiology and identifying a set of multi-modal metrics that can better describe the detrimental effects on cognitive functions of altered vascular function (Table 1). The possibility of translating these metrics into a pre-clinical setting is of utmost importance. In fact, once we have identified the biomarkers of interest in an experimental model of cognitive decline, we can explore the underlying pathophysiological processes with tools unavailable in a clinical setting.

One last important perspective in the development of imaging techniques and biomarkers is to take into account the ongoing revolution in the collaboration between artificial intelligence and medical science. To obtain the maximum information from the data collected, it is fundamentally important to design imaging protocols and pipeline analysis suitable for advanced ML approaches, which are capable of generating knowledge by extracting patterns and features that are otherwise non intelligible. It is worth noting that these systems do not have the aim of replacing the clinician, but have the fundamental role of extracting more information from data that is otherwise non readily available, and supporting the clinician work through all decisional stages.

## Figures and Tables

**Table 1:** Summary of main MRI techniques and their application to cerebrovascular diseases. In the left column, there is a brief description of the potential application of the imaging technique; in the right column, there is a brief description of the methodology.

Structural Imaging			
***Brain Segmentation and Cortex Parcellation***	Through brain segmentation and cortex parcellation, we can measure the morphology of different brain structures as well as the extension and thickness of different cortex areas.	Usually performed through dedicated software, it can leverage multi-modal inputs. Different strategies of segmentation can be implemented, from multi-atlas to deep learning.	[7,8]
**Voxel-Based Morphometry**	VBM is a technique to perform group analyses regarding the shape and density of the brain cortex	To perform VBM, it is necessary to co-register all scans in the exam to a common atlas in a standard space, then voxel-wise statistical analysis is performed.	[10,11]
Diffusion Tensor Imaging			
**Tract Based Spatial Statistics**	TBSS is a technique to perform group analyses regarding the diffusion parameters in the white matter.	After a first step of co-registration and skeletonization of the white matter, diffusion measures are projected on the WM skeleton, then voxel-wise statistical analysis is performed.	[30]
**Fiber Tracking**	Fiber tracking let us reconstruct the connections from the model of diffusion, tracking regions of coherent directions of diffusion.	After the diffusion model fitting, we can track fibers either from a seed point or with a whole brain approach. After fiber reconstruction we can extract diffusion measures along the fibers or map the connection between different brain areas.	[28,29]
Functional MRI			
**Seed Based Connectivity**	Seed based connectivity leverages a priori knowledge of the clinicians to investigate connections with a ROI of choice.	Once the chosen ROI is traced on the scan, regions of high functional connectivity can be extracted by signal timecourse similarity.	[40]
**Independent Component Analysis**	ICA extracts different functional networks expressing high timecourse similarity from a fMRI scan	ICA extracts various independent components representing the functional connectivity in the brain, both at the single subject or group analysis level. Once the networks of interest have been highlighted, it is possible confront the FC between different groups of patients.	[44]
**Graph Analysis**	Functional networks are built by representing the brain as a collection of nodes corresponding to different areas.	Once the brain connectivity is represented as a matrix of connections, it is possible to extract various mathematical measures that represent both the synchronicity and the paths of connections in the brains	[45,46]
Cerebrovascular Imaging			
**Time-of-Flight Angiography**	TOF Angiography lets us image the cerebrovascular tree to evaluate injuries or defects of microcirculation.	With the great advantage of contrast-free angiography, we can image and then reconstruct the major vessels irrorating the brain, segment them, and evaluate their size and shape.	[54,55]
**Arterial Spin Labeling**	ASL is a technique to investigate brain perfusion without the use of any contrast.	Leveraging magnetic tagging, it is possible to quantitatively measure the brain perfusion obtaining the volume of flowing blood per kg of tissue per minute in each voxel of the image	[60]
